# The minimal clinically important difference of the Participation Measurement Scale in chronic stroke

**DOI:** 10.4102/sajp.v81i1.1999

**Published:** 2025-01-28

**Authors:** Oyéné Kossi, Soraia M. Silva, Francesco Lena, Mendinatou Agbetou, Thierry Adoukonou, Peter Feys, Félix Nindorera

**Affiliations:** 1 National School of Public Health and Epidemiology, University of Parakou, Parakou, Benin; 2 Postgraduate Program in Rehabilitation Sciences, Universidade Nove de Julho (UNINOVE), São Paulo, Brazil; 3 Department of Neurology, Neuromed – Istituto Neurologico Mediterraneo, Pozzilli, Italy; 4 Department of Neurology, Faculty of Medicine, University of Parakou, Parakou, Benin; 5 Rehabilitation Research Center, Faculty of Rehabilitation Sciences, Hasselt University, Parakou, Benin; 6 National Center of Reference in Physical Therapy and Medical Rehabilitation, University Hospital Roi-Khaled, Bujumbura, Burundi

**Keywords:** chronic stroke, participation, measurement, scale, PM-Scale, minimal clinically important difference

## Abstract

**Background:**

The Participation Measurement Scale (PM-Scale) is an International Classification of Functioning, Disability and Health-based and Rasch-built scale developed specifically to assess participation in people with stroke.

**Objectives:**

Our study aimed to estimate the minimal clinically important difference (MCID) for the PM-Scale.

**Method:**

We performed a secondary analysis of data from the ‘Circuit walking, balance, cycling and strength training’ trial. Participants underwent mixed and collective physical activities or sociocultural activities for 12 weeks, and participation data were collected before and after the interventions. The activity limitations (ACTIVLIM)-Stroke scale was used as the anchor of importance. The MCID for the PM-Scale was estimated using receiver operating characteristic (ROC) curves and the Youden index.

**Results:**

Data were collected from 46 people with chronic stroke, of which 22% were female, with median (Percentile 25, Percentile 75) age of 54 (44; 60) years, and time since stroke is 24 (11; 37) months. For all participants, the PM-Scale measures range from –2.98 logits to 5.02 logits. The area under the curve (AUC) for the receiver operating characteristic (ROC)-analysis was 0.74 yielding an estimated MCID of 1.98 logit for the PM-Scale.

**Conclusion:**

Our study estimated the MCID of the PM-Scale at 1.98 logit, enabling a more precise interpretation of the outcome in the clinical and research settings.

**Clinical implications:**

An improvement of at least 1.98 logit on the PM-Scale is required to induce a clinical change in the independence in activities of daily living in people with chronic stroke.

## Introduction

The World Health Organization (WHO) defined participation as ‘an individual’s involvement in life situation’ (WHO 2001). In addition, the Convention on the Rights of Persons with Disabilities emphasises that rehabilitation services and programmes should enable persons with disabilities to attain and maintain full participation in all life aspects in order to support their human rights and dignity (World Health Organization & Bank 2011). Therefore, participation is recognised as an important component of an individual’s functioning. In particular, over the past two decades, there is an increasing interest in research on rehabilitation interventions regarding participation because of associations with life satisfaction, depressive symptoms and health-related quality of life (Erler, Kew & Juengst 2020; Goverover et al. 2017; Kossi et al. 2019). As a consequence, there is a growing focus on the development of valid and reliable participation related outcome measures (OM) serving as guiding principles through rehabilitation (Kossi et al. 2018, 2020; Pereira et al. 2023; Van de Velde et al. 2017).

Several instruments have been dedicated to the assessment of participation (Kossi et al. 2020, 2024b). For the assessment of participation following stroke in the African context Kossi et al. (2018) developed and validated the Participation Measurement Scale (PM-Scale). The PM-Scale is a Rasch-built 22-item questionnaire based on the conceptual framework of the International Classification of Functioning, disability and health (Kossi et al. 2018; Pereira et al. 2021). The PM-Scale is a face-to-face interview-based questionnaire where responders are asked to provide their perceived participation on a 3-level scale as not at all (score = 0), weakly (score = 1), or strongly (score = 2). As a Rasch-built scale, the total raw score of the PM-Scale (range: 0 to 44) can be converted into linear measure in logit (range: ‒6.56 to 6.51 logits) (Kossi et al. 2018). Higher measures indicate a higher participation. Recently, Pereira et al. (2023) translated and adapted a Brazilian version of the PM-Scale. Therefore, the PM-Scale is valid for use cross-culturally, both in Africa and in Brazil. This scale presents good psychometric properties including unidimensionality, reliability, invariance, linearity, and responsiveness. Although responsiveness is an important psychometric property of any OM, this is not ideal regarding the clinical meaning of the change in the OM as it provides only the information that the change has exceeded measurement error and variability in patients who improved. However, the minimal clinically important difference (MCID) is more useful clinically as it provides an index of important change. The MCID may involve an external anchor-based approach to estimate how much change in an OM is clinically important and meaningful. Our study aimed at determining the MCID of the PM-Scale in people with chronic stroke using three external anchors.

## Research methods and design

### Study design and setting

This is a secondary data analysis using data collected during the *‘Circuit walking, balance, cycling and strength training’ (CBCS)* trial conducted in a community facility attached to the National Center of Reference in Physical Therapy and Medical Rehabilitation, University Hospital Roi-Khaled, Bujumbur. The CBCS trial was a two-arm, parallel-group, crossover and single-blind randomised controlled trial (Nindorera et al. 2023).

### Participants and inclusion criteria

To be enrolled in our study, patients had to meet the following criteria: (1) unilateral stroke that occurred at least 6 months earlier, (2) age ≥ 18 years, and (3) ability to walk 10 m with or without an assistive device. People with major cognitive impairments or permanent degenerative damage (e.g. Parkinson’s disease, Alzheimer’s disease, etc.) were excluded (Nindorera et al. 2023).

### Interventions

Participants were randomised into 1 of 2 treatment arms: CBCS (intervention group) or sociocultural activities group (control group). Both CBCS and sociocultural interventions were performed 2 h/day, 3 times a week for 12 weeks (36 sessions: 72 h). See the study on the CBCS (Nindorera et al. 2023) for more details on the intervention dosage.

### Outcome measures and anchors

For our study, we considered data from baseline and immediately post-intervention (3 months). In the literature, multiple approaches are used for determining the MCID of OM (Wells et al. 2001), the two main being distribution-based and anchor-based approaches (Crosby, Kolotkin & Williams 2003).

Distribution based approach uses calculation of standard error of measurement and effect size to estimate MCID while the anchor-based methods compare the changes in patients’ score with another external measure of change labelled as anchor, which can be either subjective or objective (Lydick & Epstein 1993; Rai et al. 2015).

In our study, we used the anchor-based approach considering three different external anchors that were administered at each assessment time point: the modified Rankin Scale (mRS), the depression subscale of the Hospital Anxiety and Depression Scale (HADS-D), and the activity limitations (ACTIVLIM)-Stroke scale. These three anchors were chosen to provide external criteria related to impairments (HADS-D), and ACTIVLIM-Stroke, and to acquire two perspectives, one from the clinician through mRS and another from the participant through the ACTIVLIM-Stroke and HADS-D.

### Statistical analyses

Participants were dichotomised into two groups on the basis of their having achieved or not achieved important improvement after completion of the intervention. Independent t tests or non-parametric Mann Whitney tests, and Spearman ρ or Pearson r correlation analyses were performed as appropriate to compare changes and to explore the relationship between the anchors and change in PM-Scale measures. A statistically significant difference in change in PM-Scale measure between the improved and stable/not improved groups and statistically significant relationship between anchor and the outcome of interest were required to support the validity of the anchors (Fulk & He 2018). The sensitivity of the PM-Scale was calculated using receiver operating characteristic (ROC) curves. The area under the curve (AUC) was used to describe the ability of the PM-Scale measure to distinguish patients who improved from those who did not improve. The AUC was insufficient at ˂ 0.7, acceptable from 0.7 to 0.8, and excellent at ≥ 0.8 (Cronbach 1951). The optimal MCID of the PM-Scale measure was subsequently determined using Younden index. Statistical analyses were performed using RStudio software (RStudio 2023.06.1 + 524 version 4.2.2, R Foundation for Statistical Computing) with glm, tidyr and pROC packages. A *p*-value under 0.05 was set as statistically significant.

### Ethical considerations

Our study received approval from the local Ethics Committee (approval n: CNE/25/2019), and the protocol was registered in the Pan African Clinical Trials Registry (https://pactr.samrc.ac.za/; no. PACTR202001714888482). All individual participants gave informed written consent prior to inclusion in our study.

## Results

Data were collected from 46 people with chronic stroke, of which 22% were females, with a median (Percentile 25, Percentile 75) age of 54 (44; 60) years, and time since stroke 24 (11; 37) months. For all participants, the PM-Scale measures range from −2.98 logits to 5.02 logits. Specifically, the PM-Scale measure at enrolment was 0.61(−0.02; 1.44) logits, and immediately post-intervention was 2.49 (1.44; 3.42) logits with a mean change pre-post intervention of 1.76 (1; 2.77) logits. Data across the three anchors are presented in Table 1 and Table 2 for the collective physical activity intervention and in Table 3 for the sociocultural intervention.

**TABLE 1:** Participation measurement within the community-based collective physical activity intervention.

Variable	All participants		HADS-D				Activity limitations -Stroke				mRS			
			Improved (*n* = 21)		Not improved (*n* = 2), indiv. value		Improved (*n* = 10)		Not improved (*n* = 13)		Improved (*n* = 19)		Not improved (*n* = 4)	
	*n*	Median (P25; P75)	*n*	Median (P25; P75)	*n*	Median (P25; P75)	*n*	Median (P25; P75)	*n*	Median (P25; P75)	*n*	Median (P25; P75)	*n*	Median (P25; P75)
Age (years)	54.00	44.00; 60.00	54.00	44.00; 57.00	-	47.00; 69.00	57.00	44.00; 63.00	47.00	43.00; 55.00	47.00	44.00; 60.00	55.50	46.00; 63.00
Time since stroke (months)	24.00	11.00; 37.00	24.00	11.00; 36.00	-	48.00; 69.00	27.00	11.00; 37.00	24.00	11.00; 37.00	24.00	11.00; 37.00	38.00	19.50; 53.00
PM-Scale measures: T0 (logit)	0.61	−0.02; 1.44	0.41	−0.02; 1.38	-	0.62; 3.00	0.82	−0.02; 1.44	0.41	−0.02; 1.29	0.41	−0.02; 1.44	0.95	0.30; 1.87
PM-Scale measures: T1 (logit)	2.49	1.44; 3.42	2.35	1.44; 3.42	-	2.49; 2.49	2.95	1.44; 3.74	2.14	1.37; 2.85	2.41	1.37; 3.42	3.14	2.67; 4.18
PM-Scale: change between T0 and T1 (logit)	1.76	1.00; 2.77	1.76	1.24; 2.77	-	−0.51; 1.87	2.11	1.76; 3.08	1.46	1.00; 2.20	1.68	0.97; 2.77	2.18	1.71; 2.97

P, percentile; mRS, modified Rankin scale; HADS-D, depression subscale of the hospital anxiety and depression scale; T0, immediately pre-intervention; T1, immediately post-intervention, indiv, individual; PM-Scale, Participation Measurement Scale.

**TABLE 2:** Pre-post intervention of Participation Measurement Scale measures within the community-based collective physical activity intervention.

Variable	Improved (*n* = 21)		Not improved (*n* = 2), indiv. value	
	*n*	Median (P25; P75)	*n*	Median (P25; P75)
Age median (years)	54.00	44.00; 57.00	-	47.00; 69.00
Time since stroke (months)	24.00	11.00; 37.00	-	36.00; 69.00
PM-Scale measures. T0. logit.	0.41	−0.02; 1.38	-	1.02; 3.00
PM-Scale measures. T1. logit.	2.49	1.44; 3.42	-	1.02; 2.49
PM-Scale. change between T0 & T1. logit.	1.87	1.35; 2.77	-	−0.51; 0.00

Indiv., individual; P, percentile; PM-Scale, Participation Measurement Scale.

**TABLE 3:** Participation measurement within the sociocultural intervention.

Variable	Allparticipants		HADS-D				mRS Comparison T0 & T1 (Mann Whitney test)				*p*
			Improved (*n* = 20)		Not improved (*n* = 3), indiv. value		Improved (*n* = 14)		Not improved (*n* = 9)		
	*n*	Median (P25; P75)	*n*	Median (P25; P75)	*n*	Median (P25; P75)	*n*	Median (P25; P75)	*n*	Median (P25; P75)	
Age (months)	50.00	44.00; 57.00	49.00	45.00; 57.00	41.00	50.00; 65.00	52.00	44.00; 57.00	48.00	46.00; 51.00	< 0.001
Time since stroke (months)	21.00	9.00; 32.00	16.50	8.50; 25.50	2.00	48.00; 120.00	24.00	13.00; 48.00	9.00	8.00; 24.00	< 0.001
PM-Scale measures. T0. logit.	0.19	−0.35; 1.26	0.53	−0.26; 1.57	−1.56	−0.23; 0.19	0.41	−0.02; 1.88	−0.16	−0.82; 0.92	0.370
PM-Scale measures. T1. logit.	2.11	1.23; 3.42	2.11	1.06; 3.42	1.23	1.88; 2.27	2.31	1.88; 3.42	0.83	0.33; 2.11	< 0.001
PM-Scale. change between T0 and T1. logit.	1.10	0.22; 1.82	0.99	0.22; 1.74	0.34	1.17; 2.58	1.68	0.31; 2.08	0.24	0.21; 0.87	< 0.001

P, percentile; mRS, modified Rankin scale; HADS-D, depression subscale of the hospital anxiety and depression scale; T0, immediately pre-intervention; T1, immediately post-intervention; indiv, individual.

**FIGURE 1:**
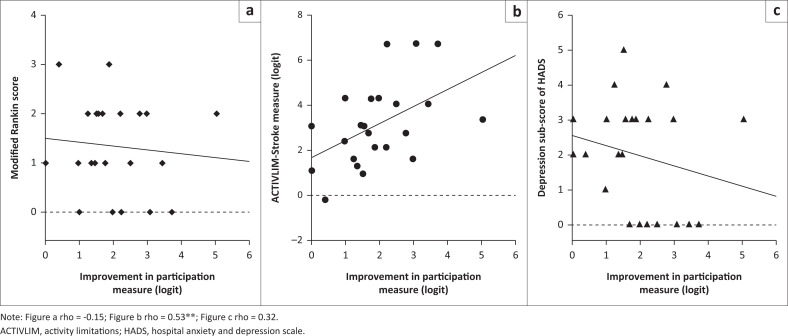
Correlations between changes in Participation Measurement Scale and the modified rankin scale (a), the activity limitations-Stroke scale (b), and the depression sub-scale (c) of the hospital anxiety and depression scale.

**FIGURE 2:**
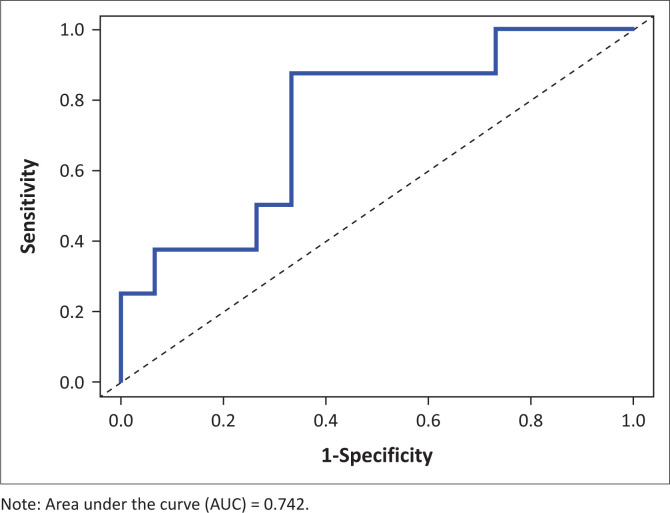
Receiver operating curves using activity limitations-Stroke measure as the anchor of importance difference.

Figure 1 shows significant, but moderate correlation between the ACTIVLIM-Stroke measure (r = 0.53, *p* < 10^−2^) and changes in PM-Scale measure, while there was no significant correlation between the mRS score and HADS score and changes in PM-Scale measure. Consequently, we did not consider the latter two anchors for further analyses for the MCID. Based on the ACTIVLIM-Stroke measure as the anchor of importance, the AUC for the ROC analysis was 0.74 (Figure 2) which resulted in an estimated MCID of 1.98 logit for the PM-Scale (Table 4).

**TABLE 4:** Estimation of the Youden index and the minimal clinically important difference of the Participation Measurement Scale based on the activity limitations-Stroke scale as anchor of importance.

Difference in ACTIVLIM-stroke measure (logit)	Clinical improvement	Difference in PM-Scale measure (logit)	Sensitivity (se)	Specificity (sp)	1 – sp	se + sp –1	(1–se)^2^ + (1–sp)^2^
3.04	Yes	5.04	0.00	0.88	0.13	−0.13	1.01
3.45	Yes	0.97	0.67	0.00	1.00	−0.33	1.11
2.92	No	0.00	1.00	0.00	1.00	0.00	1.00
2.15	No	1.46	0.60	0.13	0.88	−0.28	0.93
2.38	No	1.00	0.60	0.13	0.88	−0.28	0.93
2.02	No	1.68	0.33	0.13	0.88	−0.54	1.21
1.82	No	2.98	0.07	0.63	0.38	−0.31	1.01
3.12	Yes	1.76	0.33	0.13	0.88	−0.54	1.21
3.14	Yes	1.98†	0.67	0.38	0.63	0.04	0.50
2.38	No	1.51	0.40	0.13	0.88	−0.48	1.13
1.77	No	2.20	0.27	0.50	0.50	−0.23	0.79
5.02	Yes	3.72	0.00	0.75	0.25	−0.25	1.06
2.34	No	3.44	0.00	0.75	0.25	−0.25	1.06
2.56	No	2.50	0.20	0.63	0.38	−0.18	0.78
5.21	Yes	2.23	0.27	0.50	0.50	−0.23	0.79
2.55	No	1.24	0.60	0.13	0.88	−0.28	0.93
3.59	Yes	1.87	0.67	0.25	0.75	−0.08	0.67
1.48	No	0.40	0.73	0.00	1.00	−0.27	1.07
2.62	No	2.77	0.13	0.63	0.38	−0.25	0.89
4.31	Yes	3.08	0.07	0.63	0.38	−0.31	1.01
1.10	No	1.35	0.53	0.13	0.88	−0.34	0.98
2.14	No	1.56	0.40	0.13	0.86	−0.48	1.13
1.10	No	0.02	0.87	0.00	1.00	−0.13	1.02

MCID, minimal clinically important difference; ACTIVLIM, activity limitations; se, sensitivity; sp, specificity.

† , minimal clinically important difference.

## Discussion

Our study aimed to determine the MCID for the PM-Scale in people with chronic stroke after a community-based collective physical activity intervention utilising anchor-based approaches. Using the ACTIVLIM-Stroke scale as the anchor of importance, the AUC for the ROC-analysis was 0.74 resulting in an MCID of 1.98 logit of the PM-Scale.

The anchor-based method was used in our study to connect changes in the PM-Scale with the external anchor. Our study examined three potential anchors to calculate MCID: the mRS, the depression subscale of the HADS, and the ACTIVLIM-Stroke scale. However, the correlation between both mRS and the HADS anchors with PM-Scale was very low and non-significant (rho < 0.4). Meanwhile, the correlation between the ACTIVLIM-Stroke scale anchors with PM-Scale was acceptable and significant (r > 0.5). The occurrence of a suitable correlation indicates that the ACTIVLIM-Stroke scale was an appropriate anchor. This finding is in accordance with the result of a previous study which suggests that activities of daily life (ADLs) measured by the ACTIVLIM-Stroke scale were the strongest predictor of participation (Kossi et al. 2019).

The results of our study may be useful to clinicians in determining if a person with a chronic stroke has seen important changes in his or her social participation by assessing changes in PM-Scale measures (Kossi et al. 2024a; Nindorera et al. 2022, 2023). Minimal clinically important difference values may help assessing the effect of an intervention on a particular individual or for monitoring the change process. Clinicians may use the estimated MCID of the PM-Scale to assist when making clinical decisions regarding patient change and differences between groups (Amanzonwé et al. 2023; Kossi et al. 2024b). For example, consider the case when a patient’s initial PM-Scale measure was 0 logit at initial examination, and 1 month later, it improved to 1.5 logit (< the estimated MCID). The interpretation of this change would suggest that the patient did not experience a clinically important improvement in the PM-Scale in relation to overall independence in ADLs. However, if another patient’s PM-Scale measure improved for 2 logits (> estimated MCID), this would indicate that the patient experienced a clinically important improvement in the PM-Scale in relation to overall independence in ADLs.

In research, intra- or inter-group comparisons may be made by identifying patients with more changes. Additionally, the value of the MCID may be useful for the calculation of the sample size in interventions aiming to assess social participation in individuals with stroke using the PM-Scale as the primary outcome. Recent research suggests a paucity of evidence of rehabilitation interventions in improving participation outcomes in common neurological conditions (Kossi et al. 2024b). Possible explanation of this fact was the use of inappropriate participation OMs. As a result, there is a need for future studies to be conducted for the accurate assessment of interventions aiming at improving participation as primary outcome in neurological disorders using appropriate outcomes measures such as the PM-Scale (Kamenov et al. 2019; Kossi 2023; Kossi et al. 2020, 2024b).

In summary, the determination of the MCID for the PM-Scale will enhance the interpretation of the outcome, facilitate personalised care and support evidence-based decision-making. Indeed, the current literature shows that the MCID is highly context-specific and depends on several factors, including (1) the disease severity, (2) the outcome, (3) the patient’s treatment regimen, and (4) contextual factors (e.g., culture) (Goyal et al. 2022).

A potential limitation of our study would be the small sample size, that may limit the generalisability of the findings. In addition, the MCID value found in our study applies only within the chronic stage of stroke. Whether this estimate applies to the acute or subacute phases of stroke remains to be studied. Nevertheless, one main recommendation emerges from our findings. We recommend that researchers use the MCID of 1.98 logits found in our study for the calculation of the sample size in studies aiming at estimating the effects of rehabilitation interventions on social participation with the PM-Scale used as the primary OM.

## Conclusion

Considering the latent nature of the social participation, it is important to interpret assessments accurately. Our study provides the first estimates of MCID of the PM-Scale. The findings will allow a more precise interpretation of interventions targeting social participation in people with chronic stroke in the clinical context.
